# Supplementary material for: Comparison of hip function and quality of life of total hip arthroplasty and resurfacing arthroplasty in the treatment of young patients with arthritis of the hip joint at 5 years
Source: BMJ Open. 2018 Mar 12;8(3):e018849. doi: 10.1136/bmjopen-2017-018849 (PMC5879574; doi:10.1136/bmjopen-2017-018849)
Supplement: Supplementary file 1 [file bmjopen-2017-018849supp001.pdf]

Appendix

A.1 GEE models for OHS

Model 1: OHS = OHS.Base + Year, with identity link function and Gaussian variance to mean relation and first order autoregressive correlation structure (QIC = 53132.68).

Model 1: Parameter estimates and naïve and robust standard errors (SE)						
Term	*Estimate*	*Naive SE*	*Naive z*	*Robust SE*	*Robust z*	*p-value*
(Intercept)	27.927	2.120	13.174	2.561	10.903	0.000
OHS Base	0.637	0.096	6.655	0.106	6.007	0.000
Year	-0.701	0.267	-2.623	0.237	-2.963	0.003
Estimated Scale Parameter: 98.932; Number of Iterations: 3						

Model 1: Working correlation model for estimated correlation parameter 0.755					
Occasion	*Year 1*	*Year 2*	*Year 3*	*Year 4*	*Year 5*
Year 1	1.000	0.755	0.570	0.430	0.325
Year 2	0.755	1.000	0.755	0.570	0.430
Year 3	0.570	0.755	1.000	0.755	0.570
Year 4	0.430	0.570	0.755	1.000	0.755
Year 5	0.325	0.430	0.570	0.755	1.000

Model 2: OHS = OHS.Base + Year + Year x Treat, with identity link function and Gaussian variance to mean relation and first order autoregressive correlation structure (QIC = 53170.18).

Model 2: Parameter estimates and naïve and robust standard errors (SE)						
Term	*Estimate*	*Naive SE*	*Naive z*	*Robust SE*	*Robust z*	*p-value*
(Intercept)	28.799	2.383	12.087	2.608	11.041	0.000
OHS.Base	0.637	0.096	6.631	0.106	5.983	0.000
Year	-0.945	0.383	-2.467	0.380	-2.488	0.013
THR	-1.715	2.143	-0.800	1.771	-0.968	0.333
Year x THR	0.474	0.534	0.888	0.474	1.001	0.317
Estimated Scale Parameter: 99.365; Number of Iterations: 3<br>THR is effect of THR treatment relative to RSA = 0						

Model 2: Working correlation model for estimated correlation parameter 0.757					
Occasion	*Year 1*	*Year 2*	*Year 3*	*Year 4*	*Year 5*
Year 1	1.000	0.757	0.573	0.434	0.329
Year 2	0.757	1.000	0.757	0.573	0.434
Year 3	0.573	0.757	1.000	0.757	0.573
Year 4	0.434	0.573	0.757	1.000	0.757
Year 5	0.329	0.434	0.573	0.757	1.000

A.2 GEE models for EQ-5D

Model 1: EQ5D = EQ5D.Base + Year, with identity link function and Gaussian variance to mean relation and first order autoregressive correlation structure (QIC = 54.82).

Model 1: Parameter estimates and naïve and robust standard errors (SE)						
Term	*Estimate*	*Naive SE*	*Naive z*	*Robust SE*	*Robust z*	*p-value*
(Intercept)	0.629	0.039	16.003	0.046	13.554	0.000
OHS Base	0.436	0.060	7.324	0.075	5.820	0.000
Year	-0.027	0.010	-2.865	0.009	-3.137	0.002
Estimated Scale Parameter: 0.092; Number of Iterations: 2						

Model 1: Working correlation model for estimated correlation parameter 0.567					
Occasion	*Year 1*	*Year 2*	*Year 3*	*Year 4*	*Year 5*
Year 1	1.000	0.567	0.322	0.182	0.103
Year 2	0.567	1.000	0.567	0.322	0.182
Year 3	0.322	0.567	1.000	0.567	0.322
Year 4	0.182	0.322	0.567	1.000	0.567
Year 5	0.103	0.182	0.322	0.567	1.000

Model 2: EQ5D = EQ5D.Base + Year + Year x Treat, with identity link function and Gaussian variance to mean relation and first order autoregressive correlation structure (QIC = 57.37).

Model 2: Parameter estimates and naïve and robust standard errors (SE)						
Term	*Estimate*	*Naive SE*	*Naive z*	*Robust SE*	*Robust z*	*p-value*
(Intercept)	0.647	0.051	12.648	0.055	11.682	0.000
OHS.Base	0.441	0.059	7.515	0.073	6.076	0.000
Year	-0.016	0.014	-1.212	0.012	-1.341	0.180
THR	-0.039	0.067	-0.590	0.059	-0.673	0.501
Year x THR	-0.020	0.019	-1.084	0.017	-1.186	0.236
Estimated Scale Parameter: 0.09; Number of Iterations: 3<br>THR is effect of THR treatment relative to RSA = 0						

Model 2: Working correlation model for estimated correlation parameter 0.565					
Occasion	*Year 1*	*Year 2*	*Year 3*	*Year 4*	*Year 5*
Year 1	1.000	0.565	0.319	0.180	0.102
Year 2	0.565	1.000	0.565	0.319	0.180
Year 3	0.319	0.565	1.000	0.565	0.319
Year 4	0.180	0.319	0.565	1.000	0.565
Year 5	0.102	0.180	0.319	0.565	1.000
