# Supplementary material for: Comparison of hip function and quality of life of total hip arthroplasty and resurfacing arthroplasty in the treatment of young patients with arthritis of the hip joint at 5 years
Source: BMJ Open. 2018 Mar 12;8(3):e018849. doi: 10.1136/bmjopen-2017-018849 (PMC5879574; doi:10.1136/bmjopen-2017-018849)
Supplement: Author's manuscript [file bmjopen-2017-018849.draft_revisions.pdf]

BMJ Open is committed to open peer review. As part of this commitment we make the peer review history of every article we publish publicly available.

When an article is published we post the peer reviewers' comments and the authors' responses online. We also post the versions of the paper that were used during peer review. These are the versions that the peer review comments apply to.

The versions of the paper that follow are the versions that were submitted during the peer review process. They are not the versions of record or the final published versions. They should not be cited or distributed as the published version of this manuscript.

BMJ Open is an open access journal and the full, final, typeset and author-corrected version of record of the manuscript is available on our site with no access controls, subscription charges or pay-per-view fees (http://bmjopen.bmj.com).

If you have any questions on BMJ Open's open peer review process please email editorial.bmjopen@bmj.com

BMJ Open

WARWICK ARTHROPLASTY TRIAL: MEDIUM TERM FOLLOW-UP

Journal:	*BMJ Open*
Manuscript ID	bmjopen-2017-018849
Article Type:	Research
Date Submitted by the Author:	07-Aug-2017

Complete List of Authors:	Costa, Matthew; University of Oxford, Nuffield Department of Orthopaedics, Rheumatology, and Musculoskeletal Sciences<br>Achten, Juul; University of Oxford, NDORMS, Kadoorie Centre<br>Foguet, Pedro; University Hospitals Coventry and Warwickshire NHS Trust<br>Parsons, Nicholas; University of Warwick, Warwick Medical School

Keywords:	Total Hip Replacement, Arthroplasty, Clinical effectiveness, Randomised Clinical Trial

SCHOLARONE™
Manuscripts

For peer review only

For peer review only - http://bmjopen.bmj.com/site/about/guidelines.xhtml

WARWICK ARTHROPLASTY TRIAL: MEDIUM TERM FOLLOW-UP

A Randomised Controlled Trial of Total Hip Replacement versus Resurfacing Arthroplasty in the Treatment of Young Patients with Arthritis of the Hip Joint: minimum five year follow-up study.

Matthew L Costa[1*], Juul Achten[1], Pedro Foguet[2], Nicholas Parsons[3]

[1]NDORMS, Oxford Trauma, Kadoorie Centre, University of Oxford, John Radcliffe Hospital, Oxford, OX3 7UD
[2]University Hospitals Coventry and Warwickshire NHS Trust, Clifford Bridge Road, CV22DX
[3]Warwick Medical School, University of Warwick, Coventry, CV4 7AL

*Corresponding Author
Professor Matthew Costa,
Kadoorie Centre, NDORMS, University of Oxford, Level 3, John Radcliffe Hospital, Headley Way, Oxford, OX3 9DU.
Matthew.costa@ndorms.ox.ac.uk
01865223117

Word count: 2,952

Key words: Total Hip replacement, Arthroplasty, Clinical effectiveness, Randomised Clinical Trial

For peer review only

For peer review only - http://bmjopen.bmj.com/site/about/guidelines.xhtml

Abstract

Aim To compare the medium-term clinical effectiveness of total hip replacement and resurfacing arthroplasty.

Patients and Methods 122 patients with severe arthritis of the hip joint suitable for a resurfacing arthroplasty of the hip were randomised to either total hip replacement or resurfacing arthroplasty in this single-centre, two-arm, parallel group trial. Patients were followed up annually for a minimum of five year. The outcome measures were hip function assessed using the Oxford hip score (OHS), and health-related quality of life assessed using the EuroQol (EQ-5D).

Results 95 (78%) of the 122 original study participants provided data at 5 years. There was a small decrease in both hip function and quality of life in both groups of patients each year, during the five year follow-up period. However, there was no evidence of a significant difference between treatments group in the OHS (p = 0.822) or the EQ-5D (p = 0.501).

Conclusions We previously reported no difference in outcome in the first year after surgery. The current medium-term results also show no evidence of a difference in hip function or health-related quality of life in the five years after total hip replacement versus resurfacing arthroplasty.

Trial registration Current controlled Trials ISRCTN33354155. UKCRN 4093.

For peer review only

For peer review only - http://bmjopen.bmj.com/site/about/guidelines.xhtml

Article Summary

Strength The main strength of this trial is that it is pragmatic, with a large number of surgeons using a variety of different hip arthroplasty implants and their own preferred surgical technique.

Other strengths include the use of validated patient-reported outcome tools, a relatively large number of participants for this type of trial and the high levels of complete follow-up data.

Limitation The key limitation of this trial was that the patients themselves could not be blind to their type of hip arthroplasty.

For peer review only

For peer review only - http://bmjopen.bmj.com/site/about/guidelines.xhtml

Introduction

For older patients with severe arthritis of the hip, several designs of Total Hip Arthroplasty (THR) have shown excellent long-term results in terms of both function and value for money. [1] However, in younger more active patients, there is an approximate 50% failure rate at 25 years for traditional implants.[2] Modern THR designs may improve upon these results[3], but the search for new, more durable forms of arthroplasty continues. One option is Resurfacing Arthroplasty of the Hip (RSA). [4]

Resurfacing implants are more expensive than traditional (metal and plastic) THR designs and there are potential complications associated with RSA compared with THR – most importantly the risk of fracture of the neck of the femur. [5] However, the first clinical results showed that in selected patients, 98% of RSA implants were still functioning at five years [6]; which is as good as any of the existing THR designs [1]. Furthermore, by preserving the patient's own proximal femoral anatomy, it was suggested that RSA may provide more physiological hip movement. Early clinical outcomes indicated that RSA provides improved hip function when compared with THR.[7,8] Other studies[7,9] reported that patients having RSA had higher activity levels after the procedure and were more likely to be involved in activities such as running and heavy manual labour. However, these studies were not randomised clinical trials.

In 2012, we reported the one-year results of a randomised controlled trial to compare the clinical effectiveness of total hip arthroplasty and resurfacing arthroplasty in patients with severe arthritis of the hip. [10] There was no evidence of a difference in functional outcomes or health-related quality of life at one year. Data regarding cost effectiveness was reported separately. [11] In this report we provide the minimum five year follow-up data from the same randomised trial.

For peer review only - http://bmjopen.bmj.com/site/about/guidelines.xhtml

Patients and Methods

This was a single-centre, two-arm, parallel group, assessor-blind randomised controlled trial with 1:1 treatment allocation conducted in the UK. Full details of the protocol have been described previously. [12] A summary of the methodology follows below.

Study population In this pragmatic trial, participants were eligible if they were over 18 years of age, medically fit for an operation and suitable for a resurfacing arthroplasty – patients suitable for RSA are also suitable for THR. Patients were only excluded from the study if there was evidence that the patient would be unable to adhere to trial procedures or complete questionnaires. In order to maintain independence between observed outcomes, if a recruited patient required a contralateral hip replacement during the trial period the second hip was not included in the study.

Recruitment and randomisation of participants The trial opened in May 2007 and patients were recruited between Aug 2007 and February 2010 from hip replacement clinics in a single UK hip arthroplasty centre. Due to an error in the submission of the registration details there is a discrepancy between the dates in this manuscript and the dates in the registry, but no patients were recruited into the trial before the trial was registered on ISRCTN. Eligible patients gave written informed consent. They were randomised on a 1:1 basis to receive either a THR or a RSA.

Interventions Each patient had the allocated surgery according to the preferred technique and implants of the operating surgeon. Other peri-operative interventions, such as pre-operative protocol, prophylactic antibiotics and thrombo-prophylaxis were the same for all patients. After the operation all patients were provided with the same standardised rehabilitation plan including range-of-movement exercises followed by muscle strengthening exercises. Unless the operating surgeon specifically advised otherwise, all patients were fully weight-bearing immediately after surgery.

Outcome measurements The primary outcome measure for this medium-term follow-up study was hip function, assessed using the Oxford hip score (OHS), [13] and the secondary outcome was the EuroQol 5D (EQ-5D) health-related quality of life utility score. [14] Each outcome was collected annually by self-reported postal questionnaire. All complications related to the hip arthroplasty were recorded during the course of the trial.

Statistical analysis. Longitudinal data (time course of measurements at yearly intervals), were modelled using the generalized estimating equation (GEE) approach [15]; a first order auto-regressive

For peer review only - http://bmjopen.bmj.com/site/about/guidelines.xhtml

working correlation model was adopted to account for within-subject temporal correlations of OHS and EQ-5D outcomes, which were assumed to be approximately normally distributed. Statistical significance was assessed at the 5% level and confidence intervals of estimated model coefficients were constructed by bootstrapping. Differences in complication rates between groups were assessed using Fisher's exact test.

For peer review only

For peer review only - http://bmjopen.bmj.com/site/about/guidelines.xhtml

Results

The baseline characteristics of the patients are shown in Table 1.

Table 1. Baseline characteristics of 126 participants; data shown are means (standard deviations) [10]

Treatment	RSA (n = 60)	THR (n = 66)
Sex	F = 22 and M = 38	F = 30 and M = 36
Age (years)	56.3 (7.3)	56.6 (6.6)
BMI (kg/m^2)	28.6 (6.3)	28.7 (4.6)
Primary diagnosis osteoarthritis (%)	98	93
Oxford hip score	19.1 (8.0)	19.6 (7.8)
EQ-5D score	0.33 (0.34)	0.36 (0.33)
EQ5D VAS	56.7 (22.9)	57.8 (24.3)

Four patients never had an operation, leaving 122 in the trial. In the RSA group (n = 60); 53 participants were allocated to RSA and received RSA, and 7 were allocated to THR and received RSA**.** In the THR group (n = 62); 57 participants were allocated to THR and received THR, and 5 were allocated to RSA and received THR.

The following medium-term follow-up results are based upon the treatment received (per-protocol analysis), in contrast to the previously reported study results which were based on the allocated intervention (i.e. intention to treat analysis). The amount of missing data at each time-point is shown in Table 2. A small number of patients died during follow-up, did not respond to attempts to contact them or actively asked to be withdrawn from the study. In total, 95 (78%) of the 122 original study participants provided data at 5 years. We have no reason to believe that withdrawals or loss was related to the intervention, so we will assume for purposes of analysis that data were missing at random.

Table 2. Patient follow-up at yearly intervals post-operation

Patient Status	Year of Follow-up									
	1		2		3		4		5	
	RSA	THR	RSA	THR	RSA	THR	RSA	THR	RSA	THR
Died	0	0	0	0	1	0	1	0	1	0
Missing†	0	0	7	5	6	2	7	5	11	11
Responded‡	60	62	53	57	53	59	50	56	45	50
Withdrawn	0	0	0	0	0	1	2	1	3	1
Total	60	62	60	62	60	62	60	62	60	62

† Missing data, loss to follow-up; ‡ Patient followed-up

For peer review only - http://bmjopen.bmj.com/site/about/guidelines.xhtml

Oxford Hip Scores OHS scores at each year post-operation (from year 1 to 5) were strongly positively auto-correlated; i.e. a high OHS at year one was predictive of a high OHS in subsequent years. The OHS scores decreased over time from year 1 to year 5 (GEE z-test of regression coefficients; p = 0.003, with the regression coefficient -0.70). However, this decline was unlikely to be clinically relevant in the medium term, occurring at only approximately 0.70 OHS units per year. Figure 1 shows the temporal trends in OHS for participants from operation to year 5.

There was no evidence of a significant difference between treatments group (p = 0.822) during follow-up, and the rate of decline of OHS score did not differ between treatment groups (p = 0.317)

Health-related quality of life. There was also evidence for a significant temporal change in EQ-5D (GEE z-test of regression coefficients; p = 0.002); EQ-5D scores decreased over time from year 1 to year 5, with the regression coefficient (-0.027). However, again the decline in EQ-5D scores was relatively small at approximately 0.027 units per year

EQ-5D scores showed considerable variability during follow-up, but there was no evidence for a statistically significant difference between treatments groups (p = 0.501). The rate of decline of EQ-5D scores did not differ between treatment groups (p = 0.236)

Complications. During the five years of follow-up, one patient in the RSA group had a revision arthroplasty and three in the THR group (Table 3). Two other patients in the THR group suffered a dislocation of the hip but did not require revision surgery. One patient in the RSA group had an aspiration of the hip joint but did not require revision surgery.

Fisher's exact test provides no evidence of a statistically significant difference in revision rates between the treatment groups (p = 0.619).

Table 3. Revisions at 5 years by treatment group

	RSA	THR	Total
Revised	1	3	4
Unrevised	44	47	91
Total	45	50	95

For peer review only - http://bmjopen.bmj.com/site/about/guidelines.xhtml

Discussion

Our previously reported randomised clinical trial found no evidence of a difference in hip function between patients having total hip arthroplasty (THR) versus resurfacing arthroplasty (RSA) for severe arthritis of the hip joint during the first year following surgery. [10] This medium-term follow-up study continues to shows no difference in hip function at five years. Similarly there was no difference in health-related quality of life. The number of further complications after the first year was low in both groups, but one patient in the RSA group and three in the THR group required revision arthroplasty surgery.

Only a few randomised trials have been performed comparing total hip arthroplasty with the resurfacing technique. The first studies focused on the technical aspects of the procedure, such as the position of the implants or the amount of bone removed during the resurfacing procedure [16,17]. Three trials investigated clinical outcomes for resurfacing arthroplasty compared with a specific type of total hip arthroplasty, namely metal-on-metal total hip arthroplasty. [18-20] All of these trials showed little difference in functional outcome between the groups in the first one to two years after surgery. Each of the trials included plans to perform longer-term follow-up of the participants. However, subsequent, widely-reported concerns regarding the adverse effects of metal debris from metal-on-metal bearing surfaces have made it difficult to interpret any later results from these trials; [21,22] particularly because the functional deficits associated with adverse reactions to metal debris seemed to be greater in one group (THR) than the other (RSA). [23]

A further randomised trial, [24] looked at early muscle strength in 43 patients and found greater muscle strength deficits in the RSA group. In contrast, a trial of 80 patients with dysplastic acetabula found improved early range of movement in the RSA group, albeit with no difference in functional hip scores. [25]

The only longer-term follow-up study of resurfacing versus conventional bearing-surface THR, showed no difference between groups with regard to Oxford Hip Score or quality life. [26] More patients in the resurfacing group were involved in impact activities. However, this study contained only 24 randomised patients.

The main strength of this trial is that it is entirely pragmatic, with a relatively large number of surgeons using a variety of different hip arthroplasty implants and their own preferred surgical

For peer review only - http://bmjopen.bmj.com/site/about/guidelines.xhtml

technique. Although the patients were recruited from only one centre, the large number of surgeons involved and the variety of implants is likely to reflect practice in the wider surgical community. Other strengths include the use of validated patient-reported outcome tools, a relatively large number of participants and the high levels of complete follow-up data.

The key limitation of this trial was that the patients themselves were not blind to their type of hip arthroplasty. Patients undergoing resurfacing arthroplasty of the hip in the UK, have generally been given a different pre-operative information sheet and surgical consent from than those having a total hip arthroplasty; this reflects the existing evidence regarding the different risk/benefit profile of the two procedures.

How do the results of this trial inform the debate about resurfacing arthroplasty of the hip? This trial failed to show any evidence that resurfacing arthroplasty provides improved hip function or greater quality of life when compared with total hip arthroplasty over five years. Given the new requirements for surveillance of metal-on-metal hip replacements, [27] the higher rate of revision surgery for hip resurfacing recorded on the UK national joint registry, [28] and increased costs associated with hip resurfacing, [29] it seems increasingly difficult to justify the use of this technology. We will, however, continue to review the patients in this trial with a further report planned at a minimum of ten years.

For peer review only

Collaborators. This trial was conducted on behalf of the Young Adult Hip Arthroplasty team at University Hospitals Coventry and Warwickshire NHS Trust: Matt Costa, Pedro Foguet, Udai Prakash, Damian Griffin, Richard King, Steve Krikler and Gavin Pereira.

Acknowledgements This trial was conducted on behalf of the Young Adult Hip Arthroplasty team at University Hospitals Coventry and Warwickshire NHS Trust: Matt Costa, Pedro Foguet, Udai Prakash, Damian Griffin, Richard King, Steve Krikler and Gavin Pereira. We would like to thank Becky Kearney, Katie McGuinness, Helen Richmond, Phil Jones, Kate Dennison, Zoe Buckingham, Troy Douglin and Catherine Richmond for their invaluable assistance in recruitment and data collection for this trial. We would like to thank Chris McCarthy, Chris Bridle, Ceri Jones, Tim Friede and Steve Krikler for their clinical, trials and regulatory expertise in the TSC and DMC for this trial. Finally we would like to thanks all the patients for the time and effort they gave to this trial.

This trial was co-sponsored by the University of Warwick and University Hospitals Coventry and Warwickshire NHS trust.

Ethical Approval This study has been reviewed by the Coventry Research Ethics Committee under reference number 07/Q2802/26 - approval was awarded on the 9th of May 2007. Further approval was obtained from the research & development department of UHCW NHS trust. The research carried out is in compliance with the Helsinki Declaration and all patient provided informed consent prior to be included into the study.

The work described in this manuscript has been funded through the Research for Patient Benefit scheme of the NIHR. This manuscript presents independent research commissioned by the National Institute of Health Research. The views expressed are those of the authors and not necessarily those of the NHS, the NIHR or the Department of Health.

Trial registration Current controlled Trials ISRCTN33354155. UKCRN 4093.

Contributorship statement

MC wrote the paper,
JA contributed to writing the paper and management of data collection,
PF contributed to writing the paper,
NP performed the statistical analysis and contributed to writing the paper.

Competing interests

There are no competing interests to declare.

Funding statement

This work was funded by the National of Institute Health Research, Research for Patient benefit (RfPB) under grant number PB-PG-0706-10080.

Data sharing statement

**Will individual participant data be available (including data dictionaries)?**	Yes

For peer review only

**What data in particular will be shared?**	Individual participant data that underlie the results reported in this article, after deidentification (text, tables, figures and appendices)
**What other documents will be available?**	Study protocol
**When will data be available (start and end dates)?**	Beginning 9 months and ending 36 months after article publication
**With whom?**	Investigators whose proposed use of the data has been approved by an independent review committee ("learned intermediary") identified for this purpose
**For what types of analyses?**	For individual participant data meta-analysis
**By what mechanism will data be made available?**	Proposals may be submitted up to 36 months following article publication, via the corresponding author

For peer review only

For peer review only - http://bmjopen.bmj.com/site/about/guidelines.xhtml

References

1. Murray DW, Carr AJ, Bulstrode CJ. Which primary total hip replacement? *The Journal of bone and joint surgery British volume.* 1995;77(4):520-527.
2. Wroblewski BM, Fleming PA, Siney PD. Charnley low-frictional torque arthroplasty of the hip. 20-to-30 year results. *The Journal of bone and joint surgery British volume.* 1999;81(3):427-430.
3. Digas G, Karrholm J, Thanner J, Malchau H, Herberts P. The Otto Aufranc Award. Highly cross-linked polyethylene in total hip arthroplasty: randomized evaluation of penetration rate in cemented and uncemented sockets using radiostereometric analysis. *Clinical orthopaedics and related research.* 2004(429):6-16.
4. Australian National Joint Replacement Registry. *Demographics of Hip & Knee Arthroplasty.* Adelaide: http://www.dmac.adelaide.edu.au/;2010.
5. Cuckler JM. The optimal metal-metal arthroplasty is still a total hip arthroplasty: in the affirmative. *J Arthroplasty.* 2006;21(4 Suppl 1):74-76.
6. Treacy RB, McBryde CW, Pynsent PB. Birmingham hip resurfacing arthroplasty. A minimum follow-up of five years. *The Journal of bone and joint surgery British volume.* 2005;87(2):167-170.
7. Pollard TC, Baker RP, Eastaugh-Waring SJ, Bannister GC. Treatment of the young active patient with osteoarthritis of the hip. A five- to seven-year comparison of hybrid total hip arthroplasty and metal-on-metal resurfacing. *The Journal of bone and joint surgery British volume.* 2006;88(5):592-600.
8. Lingard EA, Muthumayandi K, Holland JP. Comparison of patient-reported outcomes between hip resurfacing and total hip replacement. *The Journal of bone and joint surgery British volume.* 2009;91(12):1550-1554.
9. Vail TP, Mina CA, Yergler JD, Pietrobon R. Metal-on-metal hip resurfacing compares favorably with THA at 2 years followup. *Clinical orthopaedics and related research.* 2006;453:123-131.
10. Costa ML, Achten J, Parsons NR, et al. Percutaneous fixation with Kirschner wires versus volar locking plate fixation in adults with dorsally displaced fracture of distal radius: randomised controlled trial. *Bmj.* 2014;349:g4807.
11. Tubeuf S, Yu G, Achten J, et al. Cost effectiveness of treatment with percutaneous Kirschner wires versus volar locking plate for adult patients with a dorsally displaced fracture of the distal radius: analysis from the DRAFFT trial. *The bone & joint journal.* 2015;97-B(8):1082-1089.
12. Costa ML, Achten J, Parsons NR, et al. UK DRAFFT - a randomised controlled trial of percutaneous fixation with kirschner wires versus volar locking-plate fixation in the treatment of adult patients with a dorsally displaced fracture of the distal radius. *BMC musculoskeletal disorders.* 2011;12:201.
13. Dawson J, Fitzpatrick R, Carr A, Murray D. Questionnaire on the perceptions of patients about total hip replacement. *The Journal of bone and joint surgery British volume.* 1996;78(2):185-190.
14. Brooks R. EuroQol: the current state of play. *Health Policy.* 1996;37(1):53-72.
15. Hardin J, Hilbe J. *Generalized Estimating Equations.* London: Chapman and Hall/CRC; 2012.
16. Loughead JM, Chesney D, Holland JP, McCaskie AW. Comparison of offset in Birmingham hip resurfacing and hybrid total hip arthroplasty. *The Journal of bone and joint surgery British volume.* 2005;87(2):163-166.

For peer review only - http://bmjopen.bmj.com/site/about/guidelines.xhtml

17. Vendittoli PA, Lavigne M, Girard J, Roy AG. A randomised study comparing resection of acetabular bone at resurfacing and total hip replacement. *The Journal of bone and joint surgery British volume.* 2006;88(8):997-1002.
18. Garbuz DS, Tanzer M, Greidanus NV, Masri BA, Duncan CP. The John Charnley Award: Metal-on-metal hip resurfacing versus large-diameter head metal-on-metal total hip arthroplasty: a randomized clinical trial. *Clinical orthopaedics and related research.* 2010;468(2):318-325.
19. Lavigne M, Therrien M, Nantel J, Roy A, Prince F, Vendittoli PA. The John Charnley Award: The functional outcome of hip resurfacing and large-head THA is the same: a randomized, double-blind study. *Clinical orthopaedics and related research.* 2010;468(2):326-336.
20. Smolders JM, Hol A, Rijnberg WJ, van Susante JL. Metal ion levels and functional results after either resurfacing hip arthroplasty or conventional metal-on-metal hip arthroplasty. *Acta Orthop.* 2011;82(5):559-566.
21. Bisseling P, Smolders JM, Hol A, van Susante JL. Metal ion levels and functional results following resurfacing hip arthroplasty versus conventional small-diameter metal-on-metal total hip arthroplasty; a 3 to 5year follow-up of a randomized controlled trial. *J Arthroplasty.* 2015;30(1):61-67.
22. Vendittoli PA, Riviere C, Roy AG, Barry J, Lusignan D, Lavigne M. Metal-on-metal hip resurfacing compared with 28-mm diameter metal-on-metal total hip replacement: a randomised study with six to nine years' follow-up. *The bone & joint journal.* 2013;95-B(11):1464-1473.
23. Skinner J, Kay P. Commentary: Metal on metal hips. *Bmj.* 2011;342:d3009.
24. Jensen C, Aagaard P, Overgaard S. Recovery in mechanical muscle strength following resurfacing vs standard total hip arthroplasty - a randomised clinical trial. *Osteoarthritis Cartilage.* 2011;19(9):1108-1116.
25. Wang Q, Zhang XL, Chen YS, Shen H, Shao JJ. Resurfacing arthroplasty for hip dysplasia: a prospective randomised study. *The Journal of bone and joint surgery British volume.* 2012;94(6):768-773.
26. Haddad FS, Konan S, Tahmassebi J. A prospective comparative study of cementless total hip arthroplasty and hip resurfacing in patients under the age of 55 years: a ten-year follow-up. *The bone & joint journal.* 2015;97-B(5):617-622.
27. Carr A. Follow-up of patients with metal-on-metal hip replacements. *BMJ* 2017;358:j3387.
28. Board NJR. *Annual Joint Registry Report.* National Joint Registry;2016.
29. Clarke A, Pulikottil-Jacob R, Grove A, et al. Total hip replacement and surface replacement for the treatment of pain and disability resulting from end-stage arthritis of the hip (review of technology appraisal guidance 2 and 44): systematic review and economic evaluation. *Health technology assessment.* 2015;19(10):1-668, vii-viii.

For peer review only

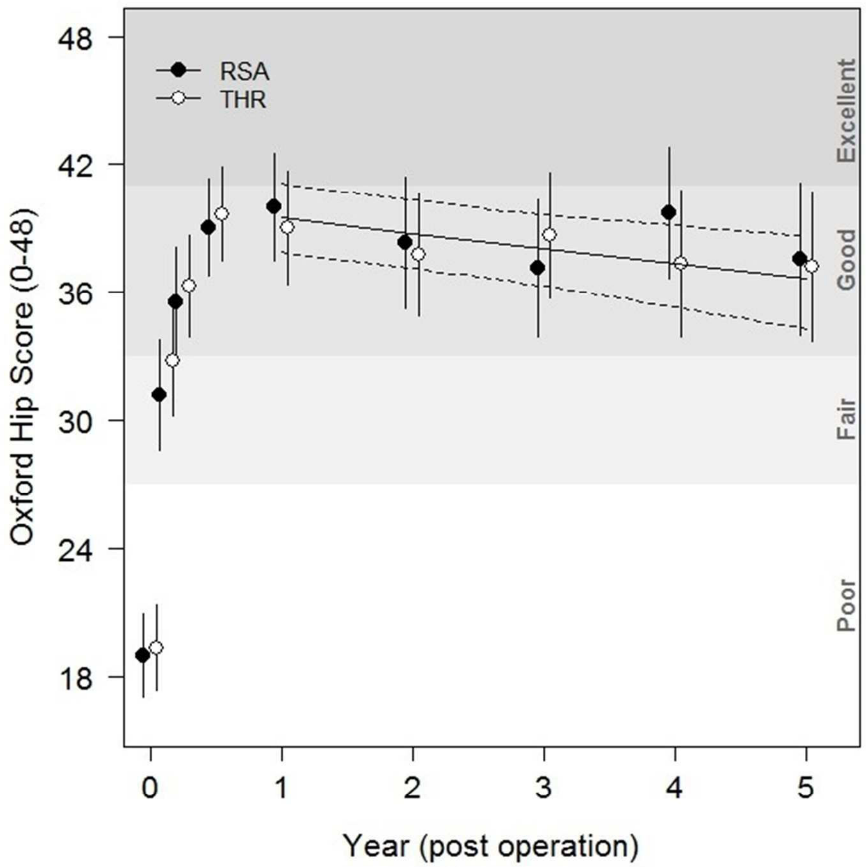

Figure 1. Temporal trends in OHS for study participants from operation to year 5; means and 95% confidence intervals for observed data, and the line indicates long term trend in scores and dashed lines bootstrapped 95% CI on trend

177x177mm (96 x 96 DPI)

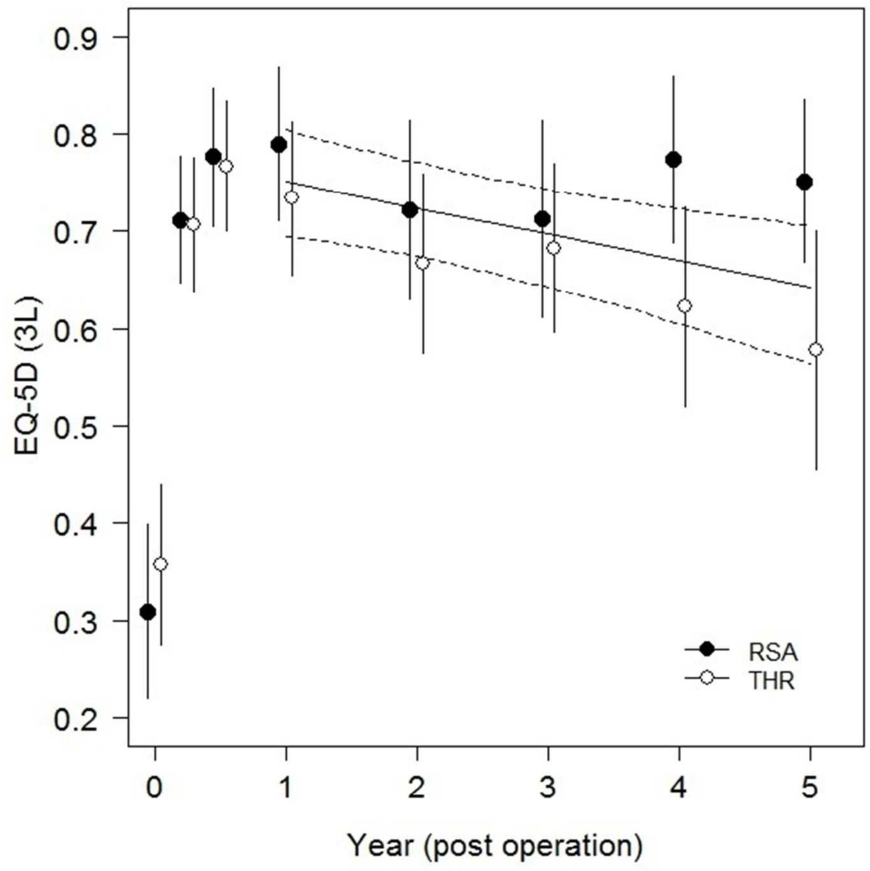

Figure 2. Temporal trends in EQ-5D (3L) score for WAT study participants from operation to year 5; means and 95% confidence intervals for observed data, and the line indicates long term trend in scores and dashed lines bootstrapped 95% CI on trend

177x177mm (96 x 96 DPI)

CONSORT 2010 checklist of information to include when reporting a randomised trial*

Section/Topic	Item No	Checklist item	Reported on page No
**Title and abstract**			
	1a	Identification as a randomised trial in the title	1
	1b	Structured summary of trial design, methods, results, and conclusions (for specific guidance see CONSORT for abstracts)	2&3
**Introduction**			
Background and objectives	2a	Scientific background and explanation of rationale	4
	2b	Specific objectives or hypotheses	4
**Methods**			
Trial design	3a	Description of trial design (such as parallel, factorial) including allocation ratio	5
	3b	Important changes to methods after trial commencement (such as eligibility criteria), with reasons	n/a
Participants	4a	Eligibility criteria for participants	5
	4b	Settings and locations where the data were collected	5
Interventions	5	The interventions for each group with sufficient details to allow replication, including how and when they were actually administered	5
Outcomes	6a	Completely defined pre-specified primary and secondary outcome measures, including how and when they were assessed	5
	6b	Any changes to trial outcomes after the trial commenced, with reasons	n/a
Sample size	7a	How sample size was determined	n/a
	7b	When applicable, explanation of any interim analyses and stopping guidelines	5-6
Randomisation:			
Sequence generation	8a	Method used to generate the random allocation sequence	5
	8b	Type of randomisation; details of any restriction (such as blocking and block size)	5
Allocation concealment mechanism	9	Mechanism used to implement the random allocation sequence (such as sequentially numbered containers), describing any steps taken to conceal the sequence until interventions were assigned	n/a
Implementation	10	Who generated the random allocation sequence, who enrolled participants, and who assigned participants to interventions	n/a
Blinding	11a	If done, who was blinded after assignment to interventions (for example, participants, care providers, those	n/a

For peer review only

		assessing outcomes) and how	
	11b	If relevant, description of the similarity of interventions	n/a
Statistical methods	12a	Statistical methods used to compare groups for primary and secondary outcomes	5-6
	12b	Methods for additional analyses, such as subgroup analyses and adjusted analyses	5-6
**Results**			
Participant flow (a diagram is strongly recommended)	13a	For each group, the numbers of participants who were randomly assigned, received intended treatment, and were analysed for the primary outcome	7
	13b	For each group, losses and exclusions after randomisation, together with reasons	7
Recruitment	14a	Dates defining the periods of recruitment and follow-up	n/a
	14b	Why the trial ended or was stopped	n/a
Baseline data	15	A table showing baseline demographic and clinical characteristics for each group	7
Numbers analysed	16	For each group, number of participants (denominator) included in each analysis and whether the analysis was by original assigned groups	7
Outcomes and estimation	17a	For each primary and secondary outcome, results for each group, and the estimated effect size and its precision (such as 95% confidence interval)	7-8
	17b	For binary outcomes, presentation of both absolute and relative effect sizes is recommended	n/a
Ancillary analyses	18	Results of any other analyses performed, including subgroup analyses and adjusted analyses, distinguishing pre-specified from exploratory	n/a
Harms	19	All important harms or unintended effects in each group (for specific guidance see CONSORT for harms)	8
**Discussion**			
Limitations	20	Trial limitations, addressing sources of potential bias, imprecision, and, if relevant, multiplicity of analyses	10
Generalisability	21	Generalisability (external validity, applicability) of the trial findings	9
Interpretation	22	Interpretation consistent with results, balancing benefits and harms, and considering other relevant evidence	9-10
**Other information**			
Registration	23	Registration number and name of trial registry	2&11
Protocol	24	Where the full trial protocol can be accessed, if available	N/A
Funding	25	Sources of funding and other support (such as supply of drugs), role of funders	11

*We strongly recommend reading this statement in conjunction with the CONSORT 2010 Explanation and Elaboration for important clarifications on all the items. If relevant, we also recommend reading CONSORT extensions for cluster randomised trials, non-inferiority and equivalence trials, non-pharmacological treatments, herbal interventions, and pragmatic trials. Additional extensions are forthcoming: for those and for up to date references relevant to this checklist, see www.consort-statement.org.

For peer review only - http://bmjopen.bmj.com/site/about/guidelines.xhtml

BMJ Open

Comparison of hip function and quality of life of Total Hip Arthroplasty and Resurfacing Arthroplasty in the Treatment of Young Patients with Arthritis of the Hip Joint at 5 years.

Journal:	*BMJ Open*
Manuscript ID	bmjopen-2017-018849.R1
Article Type:	Research
Date Submitted by the Author:	22-Nov-2017

Complete List of Authors:	Costa, Matthew; University of Oxford, Nuffield Department of Orthopaedics, Rheumatology, and Musculoskeletal Sciences<br>Achten, Juul; University of Oxford, NDORMS, Kadoorie Centre<br>Foguet, Pedro; University Hospitals Coventry and Warwickshire NHS Trust<br>Parsons, Nicholas; University of Warwick, Warwick Medical School

**Primary Subject Heading**:	Surgery
Secondary Subject Heading:	Surgery
Keywords:	Total Hip Replacement, Arthroplasty, Clinical effectiveness, Randomised Clinical Trial

SCHOLARONE™ Manuscripts

Comparison of hip function and quality of life of Total Hip Arthroplasty and Resurfacing Arthroplasty in the Treatment of Young Patients with Arthritis of the Hip Joint at 5 years.

Matthew L Costa[1*], Juul Achten[1], Pedro Foguet[2], Nicholas R Parsons[3]

[1]NDORMS, Oxford Trauma, Kadoorie Centre, University of Oxford, John Radcliffe Hospital, Oxford, OX3 7UD
[2]University Hospitals Coventry and Warwickshire NHS Trust, Clifford Bridge Road, CV22DX
[3]Warwick Medical School, University of Warwick, Coventry, CV4 7AL

*Corresponding Author
Professor Matthew Costa,
Kadoorie Centre, NDORMS, University of Oxford, Level 3, John Radcliffe Hospital, Headley Way, Oxford, OX3 9DU.
Matthew.costa@ndorms.ox.ac.uk
01865223117

Word count: 2,952

Key words: Total Hip Arthroplasty, Arthroplasty, Clinical effectiveness, Randomised Clinical Trial

For peer review only

For peer review only - http://bmjopen.bmj.com/site/about/guidelines.xhtml

Abstract

Objective To compare the medium-term clinical effectiveness of total hip arthroplasty and resurfacing arthroplasty.

Design Single centre, two arm, parallel group, assessor blinded, randomised controlled trial with 1:1 treatment allocation.

Setting A large teaching hospital in England.

Participants 122 patients older than 18 years with severe arthritis of the hip joint, suitable for resurfacing arthroplasty of the hip. Patients were excluded if they were considered to be unable to adhere to trial procedures or complete questionnaires

Interventions Total hip arthroplasty (replacement of entire femoral head and neck); hip resurfacing arthroplasty (replacement of the articular surface of femoral head only, femoral neck remains intact). Both procedures replaced the articular surface of the acetabulum.

Outcomes The outcome measures were hip function assessed using the Oxford hip score (OHS), and health-related quality of life assessed using the EuroQol (EQ-5D). Patients were followed up annually for a minimum of five year. Outcome data were modelled using the generalized estimating equation (GEE) methodology to explore temporal variations during follow-up.

Results 60 patients were randomly assigned to hip resurfacing arthroplasty and 62 to total hip arthroplasty. 95 (78%) of the 122 original study participants provided data at 5 years. There was a small decrease in both hip function and quality of life in both groups of patients each year, during the five year follow-up period. However, there was no evidence of a significant difference between treatments group in the OHS ($p = 0.333$) or the EQ-5D ($p = 0.501$).

Conclusions We previously reported no difference in outcome in the first year after surgery. The current medium-term results also show no evidence of a difference in hip function or health-related quality of life in the five years following a total hip arthroplasty versus resurfacing arthroplasty.

Trial registration Current controlled Trials ISRCTN33354155. UKCRN 4093.

Word count abstract 294

For peer review only - http://bmjopen.bmj.com/site/about/guidelines.xhtml

Article Summary

Strength The main strength of this trial is that it is pragmatic, with a large number of surgeons using a variety of different hip arthroplasty implants and their own preferred surgical technique.

Other strengths include the use of validated patient-reported outcome tools, a relatively large number of participants for this type of trial and the high levels of complete follow-up data.

Limitation The key limitation of this trial was that the patients themselves could not be blind to their type of hip arthroplasty.

For peer review only

For peer review only - http://bmjopen.bmj.com/site/about/guidelines.xhtml

Introduction

For older patients with severe arthritis of the hip, several designs of Total Hip Arthroplasty (THR) have shown excellent long-term results in terms of both function and value for money. [1] However, in younger more active patients, there is an approximate 50% failure rate at 25 years for traditional implants.[2] Modern THR designs may improve upon these results[3], but the search for new, more durable forms of arthroplasty continues. One option is Resurfacing Arthroplasty of the Hip (RSA). [4]

Resurfacing implants are more expensive than traditional (metal and plastic) THR designs and there are potential complications associated with RSA compared with THR – most importantly the risk of fracture of the neck of the femur. [5] However, the first clinical results showed that in selected patients, 98% of RSA implants were still functioning at five years [6]; which is as good as any of the existing THR designs [1]. Furthermore, by preserving the patient's own proximal femoral anatomy, it was suggested that RSA may provide more physiological hip movement. Early clinical outcomes indicated that RSA provides improved hip function when compared with THR.[7,8] Other studies[7,9] reported that patients having RSA had higher activity levels after the procedure and were more likely to be involved in activities such as running and heavy manual labour. However, these studies were not randomised clinical trials.

In 2012, we reported the one-year results of a randomised controlled trial to compare the clinical effectiveness THR and RSA in patients with severe arthritis of the hip. [10] There was no evidence of a difference in functional outcomes or health-related quality of life at one year. Data regarding cost effectiveness were reported separately. [11] In this report we provide the minimum five year follow-up data from the same cohort of patients randomized into the original trial.

For peer review only - http://bmjopen.bmj.com/site/about/guidelines.xhtml

Patients and Methods

This was a single-centre, two-arm, parallel group, assessor-blind randomised controlled trial with 1:1 treatment allocation conducted in the UK. Full details of the protocol have been described previously. [12] A summary of the methodology follows below.

Study population In this pragmatic trial, participants were eligible if they were over 18 years of age, medically fit for an operation and suitable for a RSA – patients suitable for RSA are also suitable for THR. Patients were only excluded from the study if there was evidence that the patient would be unable to adhere to trial procedures or complete questionnaires. In order to maintain independence between observed outcomes, if a recruited patient required a contralateral hip arthroplasty during the trial period the second hip was not included in the study.

Recruitment and randomisation of participants The Warwick Arthroplasty Trial (WAT) opened in May 2007 and 126 patients were recruited between Aug 2007 and February 2010 from hip arthroplasty clinics in a single UK hip arthroplasty centre. Eligible patients gave written informed consent. They were randomised on a 1:1 basis to receive either a THR or a RSA. Treatment allocation was determined using a computer generated, randomised number sequence and stratified by the supervising orthopaedic surgeon to balance any potential surgeon effects. After patients consented to participate in the trial, an independently administered, secure randomisation service was alerted by telephone of a new enrolment. The randomisation officer provided the surgeon's secretary with the patient's treatment allocation, thereby keeping the research associates, who consented patients and collected outcome data, blinded to the allocated treatment.

Interventions Each patient had the allocated surgery according to the preferred technique and implants of the operating surgeon. Other perioperative interventions, such as prophylactic antibiotics and thromboprophylaxis, were the same for all patients. After the operation, all patients underwent the same standardised rehabilitation plan, including range-of-movement exercises followed by muscle strengthening exercises. Unless the operating surgeon specifically advised otherwise, all patients were fully weight bearing immediately.

In a THR, the femoral head was removed along with most of the femoral neck. The femoral shaft was exposed to open up the femoral canal. The femoral component was then inserted into the canal and the articulating femoral head was placed onto the neck of the femoral component. The choice of components and bearing surfaces was left to the discretion of the operating surgeon, as per their usual clinical practice.

For peer review only

For peer review only - http://bmjopen.bmj.com/site/about/guidelines.xhtml

In a resurfacing arthroplasty, the articular surfaces of the femoral head was removed but the neck was left in situ. The femoral component (cap) was then impacted onto the patient's own femoral neck. All resurfacing arthroplasties of the hip used metal-on-metal bearing surfaces, but the choice of surgical approach, implant size, and positioning was left to the discretion of the operating surgeon.

In both forms of arthroplasty, the acetabulum is prepared and the acetabular component inserted into the socket.

Outcome measurements The primary outcome measure for this medium-term follow-up study was hip function, assessed using the Oxford hip score (OHS), [13] and the secondary outcome was the EuroQol 5D (EQ-5D) health-related quality of life utility score (HRQoL). [14] Each outcome was collected annually by self-reported postal questionnaire. All complications related to the hip arthroplasty were recorded during the course of the trial.

Upon completion of the main phase of the trial, the trial steering group recommended to remove the additional outcomes collected during main phase (Harris Hip Score, Disability Rating Index, Physical acticity level and resource use) to reduce the burden on the participants and to optimise retention rates.

Statistical analysis. Longitudinal data (i.e. the time course of measurements at yearly intervals), were modelled using generalized estimating equations (GEEs) to explore the population-averaged effects of time and operative treatment on function and HRQoL [15]. A first order auto-regressive working correlation model was adopted to account for within-subject temporal correlations of untransformed OHS and EQ-5D outcomes, which were assumed to be approximately normally distributed and related to the linear predictor using the identity link function. Statistical significance was assessed at the 5% level and confidence intervals of estimated temporal trends in OHS and EQ-5D were constructed by non-parametric bootstrapping. Models with smaller a quasilikelihood information criteria (QIC), where considered to be better descriptors of the data [15]. Differences in complication rates between groups were assessed using Fisher's exact test. All analyses were undertaken in R version 3.2.2 [16].

For peer review only - http://bmjopen.bmj.com/site/about/guidelines.xhtml

Results

Table 1. Baseline characteristics of 122 participants by received intervention; data shown are means (standard deviations) [10]

Treatment	RSA (n = 60)	THR (n = 62)
Sex	F = 24 and M = 36	F = 27 and M = 35
Age (years)	56.5 (6.9)	56.7 (7.0)
BMI (kg/m^2)	28.4 (6.2)	28.9 (4.8)
Oxford hip score	19.0 (7.7)	19.3 (7.9)
EQ-5D score	0.31 (0.35)	0.36 (0.36)
EQ5D VAS	56.1 (23.4)	57.6 (24.2)

The WAT study recruited 126 participants, 95% of whom had a primary diagnosis with osteoarthritis. Of the 126 participants recruited into the WAT study, four never had an operation, leaving 122 in total available for follow-up during the original trial and in the extended (medium-term) follow-up study that we report here. The baseline characteristics of these 122 participants are shown in Table 1.

The following medium-term follow-up results are based upon the treatment received (per-protocol analysis), in contrast to the previously reported study results which were based on the allocated intervention (i.e. intention to treat analysis). The amount of missing data at each time-point is shown in Table 2. A small number of patients died during follow-up, did not respond to attempts to contact them or actively asked to be withdrawn from the study. In total, 95 (78%) of the 122 original study participants provided data at 5 years. We have no reason to believe that withdrawals or loss was related to the intervention, so we will assume for purposes of analysis that data were missing completely at random.

Table 2. Patient follow-up at yearly intervals post-operation

Patient Status	Year of Follow-up									
	1		2		3		4		5	
	RSA	THR	RSA	THR	RSA	THR	RSA	THR	RSA	THR
Died	0	0	0	0	1	0	1	0	1	0
Missing†	0	0	7	5	6	2	7	5	11	11
Responded‡	60	62	53	57	53	59	50	56	45	50
Withdrawn	0	0	0	0	0	1	2	1	3	1
Total	60	62	60	62	60	62	60	62	60	62

† Missing data, loss to follow-up; ‡ Patient followed-up

For peer review only - http://bmjopen.bmj.com/site/about/guidelines.xhtml

Oxford Hip Scores OHS scores at each year post-operation (from year 1 to 5) were strongly positively auto-correlated; i.e. a high OHS at year one was predictive of a high OHS in subsequent years. The OHS scores decreased over time from year 1 to year 5 (GEE z-test of regression coefficients; p = 0.003, with the regression coefficient -0.70). However, this decline was unlikely to be clinically relevant in the medium term, occurring at only approximately 0.70 OHS units per year. Figure 1 shows the temporal trends in OHS for participants from operation to year 5.

Adding model terms to account for treatment groups (RSA *versus* THR) did not lead to a reduction in QIC and model parameters showed that there was no evidence of a significant difference between treatments group (p = 0.333) during follow-up, and the rate of decline of OHS score did not differ between treatment groups (p = 0.317). Full results of analyses are shown in Appendix A.1.

Health-related quality of life. There was also evidence for a significant temporal change in EQ-5D (GEE z-test of regression coefficients; p = 0.002); EQ-5D scores decreased over time from year 1 to year 5 (regression coefficient -0.027). However, again the decline in EQ-5D scores was relatively small at approximately 0.027 units per year

EQ-5D scores showed considerable variability during follow-up (Figure 2). Adding model terms to account for treatment groups did not lead to a reduction in QIC and model parameters showed that there was no evidence for a statistically significant difference between treatments groups (p = 0.501). The rate of decline of EQ-5D scores did not differ between treatment groups (p = 0.236). Full results of analyses are shown in Appendix A.2.

Complications. During the five years of follow-up, one patient in the RSA group had a revision arthroplasty and three in the THR group (Table 3). Two other patients in the THR group suffered a dislocation of the hip but did not require revision surgery. One patient in the RSA group had an aspiration of the hip joint but did not require revision surgery.

Fisher's exact test provides no evidence of a statistically significant difference in revision rates between the treatment groups (p = 0.619).

Table 3. Revisions at 5 years by treatment group

	RSA	THR	Total
Revised	1	3	4
Unrevised	44	47	91
Total	45	50	95

For peer review only - http://bmjopen.bmj.com/site/about/guidelines.xhtml

Discussion

Our previously reported randomised clinical trial found no evidence of a difference in hip function between patients having THR versus RSA for severe arthritis of the hip joint during the first year following surgery. [10] This medium-term follow-up study continues to shows no difference in hip function at five years. Similarly there was no difference in health-related quality of life. The number of further complications after the first year was low in both groups, but one patient in the RSA group and three in the THR group required revision arthroplasty surgery.

Only a few randomised trials have been performed comparing THR with the resurfacing technique. The first studies focused on the technical aspects of the procedure, such as the position of the implants or the amount of bone removed during the resurfacing procedured [17,18]. Three trials investigated clinical outcomes for resurfacing arthroplasty compared with a specific type of THR, namely metal-on-metal THR. [19-21] All of these trials showed little difference in functional outcome between the groups in the first one to two years after surgery. Each of the trials included plans to perform longer-term follow-up of the participants. However, subsequent, widely-reported concerns regarding the adverse effects of metal debris from metal-on-metal bearing surfaces have made it difficult to interpret any later results from these trials; [22,23]particularly because the functional deficits associated with adverse reactions to metal debris seemed to be greater in one group (THR) than the other (RSA). [24]

A further randomised trial, [25] looked at early muscle strength in 43 patients and found greater muscle strength deficits in the RSA group. In contrast, a trial of 80 patients with dysplastic acetabula found improved early range of movement in the RSA group, albeit with no difference in functional hip scores. [26]

The only longer-term follow-up study of resurfacing versus conventional bearing-surface THR, showed no difference between groups with regard to OHS or quality life. [27] More patients in the resurfacing group were involved in impact activities. However, this study contained only 24 randomised patients.

The main strength of this trial is that it is entirely pragmatic, with a relatively large number of surgeons using a variety of different hip arthroplasty implants and their own preferred surgical technique. Although the patients were recruited from only one centre, the large number of surgeons

For peer review only

For peer review only - http://bmjopen.bmj.com/site/about/guidelines.xhtml

involved and the variety of implants is likely to reflect practice in the wider surgical community. Other strengths include the use of validated patient-reported outcome tools, a relatively large number of participants and the high levels of complete follow-up data.

The key limitation of this trial was that the patients themselves were not blind to their type of hip arthroplasty. Patients undergoing RSA in the UK, have generally been given a different pre-operative information sheet and surgical consent from than those having a THR; this reflects the existing evidence regarding the different risk/benefit profile of the two procedures.

How do the results of this trial inform the debate about resurfacing arthroplasty of the hip? This trial failed to show any evidence that resurfacing arthroplasty provides improved hip function or greater quality of life when compared with THR over five years. Given the new requirements for surveillance of metal-on-metal hip arthroplasties, [28] the higher rate of revision surgery for RSA recorded on the UK national joint registry, [29] and increased costs associated with RSA, [30] it seems increasingly difficult to justify the use of this technology. We will, however, continue to review the patients in this trial with a further report planned at a minimum of ten years.

For peer review only - http://bmjopen.bmj.com/site/about/guidelines.xhtml

For peer review only

Collaborators. This trial was conducted on behalf of the Young Adult Hip Arthroplasty team at University Hospitals Coventry and Warwickshire NHS Trust: Matt Costa, Pedro Foguet, Udai Prakash, Damian Griffin, Richard King, Steve Krikler and Gavin Pereira.

Acknowledgements This trial was conducted on behalf of the Young Adult Hip Arthroplasty team at University Hospitals Coventry and Warwickshire NHS Trust: Matt Costa, Pedro Foguet, Udai Prakash, Damian Griffin, Richard King, Steve Krikler and Gavin Pereira. We would like to thank Becky Kearney, Katie McGuinness, Helen Richmond, Phil Jones, Kate Dennison, Zoe Buckingham, Troy Douglin and Catherine Richmond for their invaluable assistance in recruitment and data collection for this trial. We would like to thank Chris McCarthy, Chris Bridle, Ceri Jones, Tim Friede and Steve Krikler for their clinical, trials and regulatory expertise in the TSC and DMC for this trial. Finally we would like to thanks all the patients for the time and effort they gave to this trial.

This trial was co-sponsored by the University of Warwick and University Hospitals Coventry and Warwickshire NHS trust.

Ethical Approval This study has been reviewed by the Coventry Research Ethics Committee under reference number 07/Q2802/26 - approval was awarded on the 9th of May 2007. Further approval was obtained from the research & development department of UHCW NHS trust. The research carried out is in compliance with the Helsinki Declaration and all patient provided informed consent prior to be included into the study.

The work described in this manuscript has been funded through the Research for Patient Benefit scheme of the NIHR. This manuscript presents independent research commissioned by the National Institute of Health Research. The views expressed are those of the authors and not necessarily those of the NHS, the NIHR or the Department of Health.

Trial registration Current controlled Trials ISRCTN33354155. UKCRN 4093.

Authorship statement

MC/JA/NP designed the study and analysed and interpreted the trial data.

PF managed the recruitment and follow-up of the patients.

MC/JA/NP planned and wrote the first draft of the paper which was subsequently revised by all authors.

All authors read and approved the final manuscript. MC acts as guarantor of the paper.

Competing interests

There are no competing interests to declare.

Funding statement

This work was funded by the National of Institute Health Research, Research for Patient benefit (RfPB) under grant number PB-PG-0706-10080.

Data sharing statement

No additional data is available.

For peer review only - http://bmjopen.bmj.com/site/about/guidelines.xhtml

References

1. Murray DW, Carr AJ, Bulstrode CJ. Which primary total hip replacement? *The Journal of bone and joint surgery British volume.* 1995;77(4):520-527.
2. Wroblewski BM, Fleming PA, Siney PD. Charnley low-frictional torque arthroplasty of the hip. 20-to-30 year results. *The Journal of bone and joint surgery British volume.* 1999;81(3):427-430.
3. Digas G, Karrholm J, Thanner J, Malchau H, Herberts P. The Otto Aufranc Award. Highly cross-linked polyethylene in total hip arthroplasty: randomized evaluation of penetration rate in cemented and uncemented sockets using radiostereometric analysis. *Clinical orthopaedics and related research.* 2004(429):6-16.
4. Australian National Joint Replacement Registry. *Demographics of Hip & Knee Arthroplasty.* Adelaide: http://www.dmac.adelaide.edu.au/;2010.
5. Cuckler JM. The optimal metal-metal arthroplasty is still a total hip arthroplasty: in the affirmative. *J Arthroplasty.* 2006;21(4 Suppl 1):74-76.
6. Treacy RB, McBryde CW, Pynsent PB. Birmingham hip resurfacing arthroplasty. A minimum follow-up of five years. *The Journal of bone and joint surgery British volume.* 2005;87(2):167-170.
7. Pollard TC, Baker RP, Eastaugh-Waring SJ, Bannister GC. Treatment of the young active patient with osteoarthritis of the hip. A five- to seven-year comparison of hybrid total hip arthroplasty and metal-on-metal resurfacing. *The Journal of bone and joint surgery British volume.* 2006;88(5):592-600.
8. Lingard EA, Muthumayandi K, Holland JP. Comparison of patient-reported outcomes between hip resurfacing and total hip replacement. *The Journal of bone and joint surgery British volume.* 2009;91(12):1550-1554.
9. Vail TP, Mina CA, Yergler JD, Pietrobon R. Metal-on-metal hip resurfacing compares favorably with THA at 2 years followup. *Clinical orthopaedics and related research.* 2006;453:123-131.
10. Costa ML, Achten J, Parsons NR, et al. Total hip arthroplasty versus resurfacing arthroplasty in the treatment of patients with arthritis of the hip joint: single centre, parallel group, assessor blinded, randomised controlled trial. British Medical Journal. 2012;344:e2147
11. Edlin R, Tubeuf S, **Achten** J, Parsons N, Costa M.Cost-effectiveness of total hip **arthroplasty** versus resurfacing **arthroplasty**: economic evaluation alongside a clinical trial.BMJ Open. 2012 Oct 12;2(5).
12. **Achten** J, Parsons NR, Edlin RP, Griffin DR, Costa ML. A randomised controlled trial of total hip **arthroplasty** versus resurfacing **arthroplasty** in the treatment of young patients with arthritis of the hip joint. BMC Musculoskelet Disord. 2010 Jan 14;11:8.
13. Dawson J, Fitzpatrick R, Carr A, Murray D. Questionnaire on the perceptions of patients about total hip replacement. *The Journal of bone and joint surgery British volume.* 1996;78(2):185-190.
14. Brooks R. EuroQol: the current state of play. *Health Policy.* 1996;37(1):53-72.
15. Hardin J, Hilbe J. *Generalized Estimating Equations.* London: Chapman and Hall/CRC; 2012.
16. R Core Team (2015). R: A language and environment for statistical computing. R Foundation for Statistical Computing, Vienna, Austria. URL https://www.R-project.org/.
17. Loughead JM, Chesney D, Holland JP, McCaskie AW. Comparison of offset in Birmingham hip resurfacing and hybrid total hip arthroplasty. *The Journal of bone and joint surgery British volume.* 2005;87(2):163-166.

For peer review only - http://bmjopen.bmj.com/site/about/guidelines.xhtml

18. Vendittoli PA, Lavigne M, Girard J, Roy AG. A randomised study comparing resection of acetabular bone at resurfacing and total hip replacement. *The Journal of bone and joint surgery British volume.* 2006;88(8):997-1002.
18. Garbuz DS, Tanzer M, Greidanus NV, Masri BA, Duncan CP. The John Charnley Award: Metal-on-metal hip resurfacing versus large-diameter head metal-on-metal total hip arthroplasty: a randomized clinical trial. *Clinical orthopaedics and related research.* 2010;468(2):318-325.
20. Lavigne M, Therrien M, Nantel J, Roy A, Prince F, Vendittoli PA. The John Charnley Award: The functional outcome of hip resurfacing and large-head THA is the same: a randomized, double-blind study. *Clinical orthopaedics and related research.* 2010;468(2):326-336.
21. Smolders JM, Hol A, Rijnberg WJ, van Susante JL. Metal ion levels and functional results after either resurfacing hip arthroplasty or conventional metal-on-metal hip arthroplasty. *Acta Orthop.* 2011;82(5):559-566.
22. Bisseling P, Smolders JM, Hol A, van Susante JL. Metal ion levels and functional results following resurfacing hip arthroplasty versus conventional small-diameter metal-on-metal total hip arthroplasty; a 3 to 5year follow-up of a randomized controlled trial. *J Arthroplasty.* 2015;30(1):61-67.
23. Vendittoli PA, Riviere C, Roy AG, Barry J, Lusignan D, Lavigne M. Metal-on-metal hip resurfacing compared with 28-mm diameter metal-on-metal total hip replacement: a randomised study with six to nine years' follow-up. *The bone & joint journal.* 2013;95-B(11):1464-1473.
24. Skinner J, Kay P. Commentary: Metal on metal hips. *Bmj.* 2011;342:d3009.
25. Jensen C, Aagaard P, Overgaard S. Recovery in mechanical muscle strength following resurfacing vs standard total hip arthroplasty - a randomised clinical trial. *Osteoarthritis Cartilage.* 2011;19(9):1108-1116.
26. Wang Q, Zhang XL, Chen YS, Shen H, Shao JJ. Resurfacing arthroplasty for hip dysplasia: a prospective randomised study. *The Journal of bone and joint surgery British volume.* 2012;94(6):768-773.
27. Haddad FS, Konan S, Tahmassebi J. A prospective comparative study of cementless total hip arthroplasty and hip resurfacing in patients under the age of 55 years: a ten-year follow-up. *The bone & joint journal.* 2015;97-B(5):617-622.
28. Carr A. Follow-up of patients with metal-on-metal hip replacements. *BMJ* 2017;358:j3387.
29. Board NJR. *Annual Joint Registry Report.* National Joint Registry;2016.
30. Clarke A, Pulikottil-Jacob R, Grove A, et al. Total hip replacement and surface replacement for the treatment of pain and disability resulting from end-stage arthritis of the hip (review of technology appraisal guidance 2 and 44): systematic review and economic evaluation. *Health technology assessment.* 2015;19(10):1-668, vii-viii.

For peer review only - http://bmjopen.bmj.com/site/about/guidelines.xhtml

Figure legends

Figure 1 Temporal trends in Oxford Hip Score for participants from operation to year 5

Figure 2 Temporal trends in EQ-5D for participants from operation to year 5

For peer review only

For peer review only - http://bmjopen.bmj.com/site/about/guidelines.xhtml

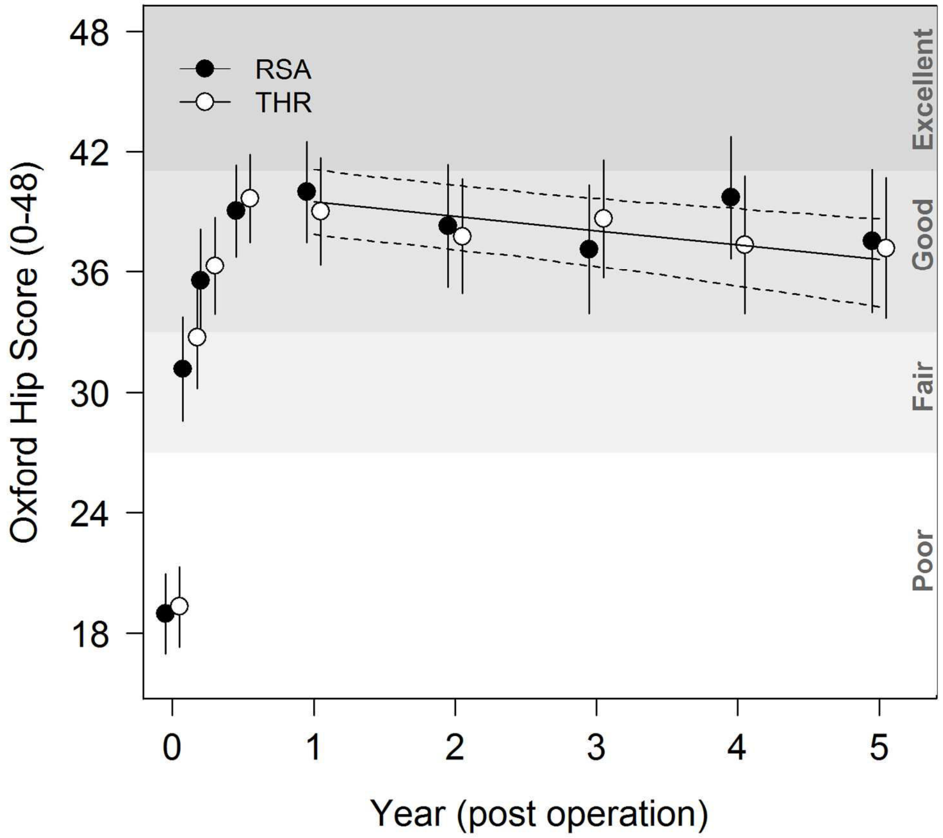

Figure 1. Temporal trends in Oxford Hip Score for WAT study participants from operation to year 5; means and 95% confidence intervals for observed data, and the line indicates long term trend in scores and dashed lines bootstrapped 95% CI on trend

152x152mm (300 x 300 DPI)

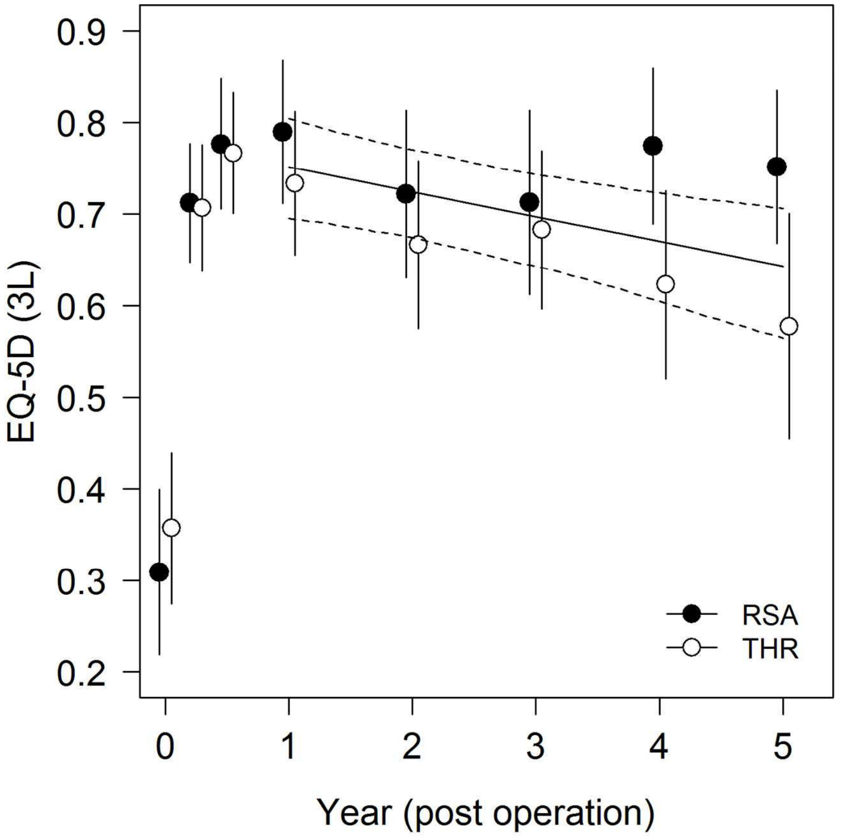

Figure 2. Temporal trends in EQ-5D (3L) score for WAT study participants from operation to year 5; means and 95% confidence intervals for observed data, and the line indicates long term trend in scores and dashed lines bootstrapped 95% CI on trend

152x152mm (300 x 300 DPI)

Appendix

A.1 GEE models for OHS

Model 1: OHS = OHS.Base + Year, with identity link function and Gaussian variance to mean relation and first order autoregressive correlation structure (QIC = 53132.68).

Model 1: Parameter estimates and naïve and robust standard errors (SE)						
Term	*Estimate*	*Naive SE*	*Naive z*	*Robust SE*	*Robust z*	*p-value*
(Intercept)	27.927	2.120	13.174	2.561	10.903	0.000
OHS Base	0.637	0.096	6.655	0.106	6.007	0.000
Year	-0.701	0.267	-2.623	0.237	-2.963	0.003
Estimated Scale Parameter: 98.932; Number of Iterations: 3						

Model 1: Working correlation model for estimated correlation parameter 0.755					
Occasion	*Year 1*	*Year 2*	*Year 3*	*Year 4*	*Year 5*
Year 1	1.000	0.755	0.570	0.430	0.325
Year 2	0.755	1.000	0.755	0.570	0.430
Year 3	0.570	0.755	1.000	0.755	0.570
Year 4	0.430	0.570	0.755	1.000	0.755
Year 5	0.325	0.430	0.570	0.755	1.000

Model 2: OHS = OHS.Base + Year + Year x Treat, with identity link function and Gaussian variance to mean relation and first order autoregressive correlation structure (QIC = 53170.18).

Model 2: Parameter estimates and naïve and robust standard errors (SE)						
Term	*Estimate*	*Naive SE*	*Naive z*	*Robust SE*	*Robust z*	*p-value*
(Intercept)	28.799	2.383	12.087	2.608	11.041	0.000
OHS.Base	0.637	0.096	6.631	0.106	5.983	0.000
Year	-0.945	0.383	-2.467	0.380	-2.488	0.013
THR	-1.715	2.143	-0.800	1.771	-0.968	0.333
Year x THR	0.474	0.534	0.888	0.474	1.001	0.317
Estimated Scale Parameter: 99.365; Number of Iterations: 3<br>THR is effect of THR treatment relative to RSA = 0						

Model 2: Working correlation model for estimated correlation parameter 0.757					
Occasion	*Year 1*	*Year 2*	*Year 3*	*Year 4*	*Year 5*
Year 1	1.000	0.757	0.573	0.434	0.329
Year 2	0.757	1.000	0.757	0.573	0.434
Year 3	0.573	0.757	1.000	0.757	0.573
Year 4	0.434	0.573	0.757	1.000	0.757
Year 5	0.329	0.434	0.573	0.757	1.000

For peer review only - http://bmjopen.bmj.com/site/about/guidelines.xhtml

A.2 GEE models for EQ-5D

Model 1: EQ5D = EQ5D.Base + Year, with identity link function and Gaussian variance to mean relation and first order autoregressive correlation structure (QIC = 54.82).

Model 1: Parameter estimates and naïve and robust standard errors (SE)						
Term	*Estimate*	*Naive SE*	*Naive z*	*Robust SE*	*Robust z*	*p-value*
(Intercept)	0.629	0.039	16.003	0.046	13.554	0.000
OHS Base	0.436	0.060	7.324	0.075	5.820	0.000
Year	-0.027	0.010	-2.865	0.009	-3.137	0.002
Estimated Scale Parameter: 0.092; Number of Iterations: 2						

Model 1: Working correlation model for estimated correlation parameter 0.567					
Occasion	*Year 1*	*Year 2*	*Year 3*	*Year 4*	*Year 5*
Year 1	1.000	0.567	0.322	0.182	0.103
Year 2	0.567	1.000	0.567	0.322	0.182
Year 3	0.322	0.567	1.000	0.567	0.322
Year 4	0.182	0.322	0.567	1.000	0.567
Year 5	0.103	0.182	0.322	0.567	1.000

Model 2: EQ5D = EQ5D.Base + Year + Year x Treat, with identity link function and Gaussian variance to mean relation and first order autoregressive correlation structure (QIC = 57.37).

Model 2: Parameter estimates and naïve and robust standard errors (SE)						
Term	*Estimate*	*Naive SE*	*Naive z*	*Robust SE*	*Robust z*	*p-value*
(Intercept)	0.647	0.051	12.648	0.055	11.682	0.000
OHS.Base	0.441	0.059	7.515	0.073	6.076	0.000
Year	-0.016	0.014	-1.212	0.012	-1.341	0.180
THR	-0.039	0.067	-0.590	0.059	-0.673	0.501
Year x THR	-0.020	0.019	-1.084	0.017	-1.186	0.236
Estimated Scale Parameter: 0.09; Number of Iterations: 3<br>THR is effect of THR treatment relative to RSA = 0						

Model 2: Working correlation model for estimated correlation parameter 0.565					
Occasion	*Year 1*	*Year 2*	*Year 3*	*Year 4*	*Year 5*
Year 1	1.000	0.565	0.319	0.180	0.102
Year 2	0.565	1.000	0.565	0.319	0.180
Year 3	0.319	0.565	1.000	0.565	0.319
Year 4	0.180	0.319	0.565	1.000	0.565
Year 5	0.102	0.180	0.319	0.565	1.000

For peer review only

For peer review only - http://bmjopen.bmj.com/site/about/guidelines.xhtml

CONSORT 2010 checklist of information to include when reporting a randomised trial*

Section/Topic	Item No	Checklist item	Reported on page No
**Title and abstract**			
	1a	Identification as a randomised trial in the title	1
	1b	Structured summary of trial design, methods, results, and conclusions (for specific guidance see CONSORT for abstracts)	2&3
**Introduction**			
Background and objectives	2a	Scientific background and explanation of rationale	4
	2b	Specific objectives or hypotheses	4
**Methods**			
Trial design	3a	Description of trial design (such as parallel, factorial) including allocation ratio	5
	3b	Important changes to methods after trial commencement (such as eligibility criteria), with reasons	n/a
Participants	4a	Eligibility criteria for participants	5
	4b	Settings and locations where the data were collected	5
Interventions	5	The interventions for each group with sufficient details to allow replication, including how and when they were actually administered	5
Outcomes	6a	Completely defined pre-specified primary and secondary outcome measures, including how and when they were assessed	5
	6b	Any changes to trial outcomes after the trial commenced, with reasons	n/a
Sample size	7a	How sample size was determined	n/a
	7b	When applicable, explanation of any interim analyses and stopping guidelines	5-6
Randomisation:			
Sequence generation	8a	Method used to generate the random allocation sequence	5
	8b	Type of randomisation; details of any restriction (such as blocking and block size)	5
Allocation concealment mechanism	9	Mechanism used to implement the random allocation sequence (such as sequentially numbered containers), describing any steps taken to conceal the sequence until interventions were assigned	n/a
Implementation	10	Who generated the random allocation sequence, who enrolled participants, and who assigned participants to interventions	n/a
Blinding	11a	If done, who was blinded after assignment to interventions (for example, participants, care providers, those	n/a

For peer review only - http://bmjopen.bmj.com/site/about/guidelines.xhtml

		assessing outcomes) and how	
	11b	If relevant, description of the similarity of interventions	n/a
Statistical methods	12a	Statistical methods used to compare groups for primary and secondary outcomes	5-6
	12b	Methods for additional analyses, such as subgroup analyses and adjusted analyses	5-6
**Results**			
Participant flow (a diagram is strongly recommended)	13a	For each group, the numbers of participants who were randomly assigned, received intended treatment, and were analysed for the primary outcome	7
	13b	For each group, losses and exclusions after randomisation, together with reasons	7
Recruitment	14a	Dates defining the periods of recruitment and follow-up	n/a
	14b	Why the trial ended or was stopped	n/a
Baseline data	15	A table showing baseline demographic and clinical characteristics for each group	7
Numbers analysed	16	For each group, number of participants (denominator) included in each analysis and whether the analysis was by original assigned groups	7
Outcomes and estimation	17a	For each primary and secondary outcome, results for each group, and the estimated effect size and its precision (such as 95% confidence interval)	7-8
	17b	For binary outcomes, presentation of both absolute and relative effect sizes is recommended	n/a
Ancillary analyses	18	Results of any other analyses performed, including subgroup analyses and adjusted analyses, distinguishing pre-specified from exploratory	n/a
Harms	19	All important harms or unintended effects in each group (for specific guidance see CONSORT for harms)	8
**Discussion**			
Limitations	20	Trial limitations, addressing sources of potential bias, imprecision, and, if relevant, multiplicity of analyses	10
Generalisability	21	Generalisability (external validity, applicability) of the trial findings	9
Interpretation	22	Interpretation consistent with results, balancing benefits and harms, and considering other relevant evidence	9-10
**Other information**			
Registration	23	Registration number and name of trial registry	2&11
Protocol	24	Where the full trial protocol can be accessed, if available	N/A
Funding	25	Sources of funding and other support (such as supply of drugs), role of funders	11

*We strongly recommend reading this statement in conjunction with the CONSORT 2010 Explanation and Elaboration for important clarifications on all the items. If relevant, we also recommend reading CONSORT extensions for cluster randomised trials, non-inferiority and equivalence trials, non-pharmacological treatments, herbal interventions, and pragmatic trials. Additional extensions are forthcoming: for those and for up to date references relevant to this checklist, see www.consort-statement.org.

For peer review only - http://bmjopen.bmj.com/site/about/guidelines.xhtml
